# Effect of resin infiltration and remineralization on color restorability and stability of artificial demineralized enamel lesions

**DOI:** 10.1186/s12903-026-08122-y

**Published:** 2026-04-02

**Authors:** Hebatallah Ali Yehia Saeed, Rana Abdel-Rehim Fouad Sedky, Eman Nasser Ahmed Diab

**Affiliations:** Operative Dentistry Department, Faculty of Dentistry, Ain Shams University, Organization of African Unity St.‑Abbasia, Cairo, 11566 Egypt

**Keywords:** Enamel demineralization, Tooth remineralization, Resin infiltration, Color restorability, Color stability

## Abstract

**Background:**

This study aimed to evaluate the effect of resin infiltration, remineralization and their combination on the color restorability of artificially acid demineralized enamel lesions in addition to the color stability of the aforementioned treatments after acid challenge and immersion in common staining beverages.

**Methods:**

A hundred and five specimens were obtained from the buccal and lingual surfaces of permanent posterior teeth. Demineralized enamel lesions were artificially induced in all samples except for positive control. All specimens were randomly divided into 5 groups (*n* = 21) according to the treatment applied to the artificially demineralized enamel lesion: sound enamel (+ ve control), demineralized enamel (-ve control), resin infiltration, remineralization using Clinpro™ White Varnish and remineralization using Clinpro™ White Varnish followed by resin infiltration. After treatment, all specimens were subjected to pH cycling daily for 28 days and immersed in one of three beverages (coffee, tea and artificial saliva) for 30 min daily. Color measurement using the Commission International de l’Eclariage (CIE) L* a* b* values, using Cary 5000 Spectrophotometer (Agilent Technologies, NY, USA) was performed three times (baseline, after treatment and after pH cycling and immersion in beverages). Results were collected and statistically analyzed.

**Results:**

The treatments applied to demineralized enamel lesions had a significant effect on color restorability (*p* < 0.001). The highest color difference values between baseline and after treatment (ΔE1) among the treatment groups was found in remineralization group (1.99 ± 0.13) while the lowest was found in remineralization & resin infiltration (0.86 ± 0.12). Regarding color stability, both treatments and beverages had a significant effect. The highest color difference values between after treatment and after pH cycling and immersion in beverages(ΔE2) was found in remineralization & resin infiltration- coffee group (9.05 ± 0.67) while the lowest values were found in the remineralization-saliva group (0.73 ± 0.12). All post hoc pairwise comparisons were statistically significant (*p* < 0.001) in all groups.

**Conclusions:**

Remineralization combined with resin infiltration can improve the esthetic outcome of demineralized enamel lesions compared to each treatment alone. However, the color stability of this combined treatment when subjected to coffee and tea is inferior to each of the treatments alone.

## Introduction

Minimally invasive dentistry is currently a major trend in modern dentistry. The main aim of minimally invasive techniques is to prevent, control and treat oral disease with maximal conservation of tooth structure [1]. Strategies of minimally invasive dentistry have largely evolved in the last few decades. Remineralization, pits and fissure sealants, preventive resin restorations, microabrasion and more recently resin infiltration have all emerged specifically for the management of non-cavitated carious lesions also known as white spot lesions (WSLs) [2, 3]. 

Active carious WSLs are usually characterized by having a chalky white, opaque appearance and are commonly located in plaque retentive areas of the teeth and around orthodontic brackets [3, 4]. The whitish appearance is the result of a decrease in the mineral content of enamel leading to a porous structure which is filled with organic fluids. The difference in refractive indices between hydroxyapatite and organic fluids causes scattering of light which leads to a whitish appearance [5, 6]. Treatment and management of WSLs is not only essential to halt the progression of caries, but also to eliminate the whitish appearance which is a cause of esthetic discomfort to patients [7]. 

Remineralization is one of the most common methods for the treatment of carious WSLs. Several remineralizing agents are present to date such as fluoride, CPP-ACP and self-assembling peptides [8]. Despite the continuous development and progression in remineralizing agents, fluoride continues to be the gold standard and the cornerstone of remineralization [9, 10]. Fluoride agents have been shown to be effective not only in preventing caries in children and adolescents but also in masking WSLs [11–13]. However, a major drawback in the fluoride-based remineralization is the limitation of mineral gain to the outer 30 μm of the lesion only [14]. This may lead to clinical visibility of remineralized lesion [15]. 

Clinpro™ White Varnish (3 M ESPE, USA) is a fluoride based remineralizing agent. It contains functionalized tricalcium phosphate(*f*TCP) in addition to 5% sodium fluoride. *f*TCP was reported to increase the efficacy of fluoride by deeper penetration into the demineralized enamel lesion and increasing availability of calcium, phosphate and fluoride thus enhancing remineralization. In addition, fumaric acid is also another component present which is claimed by the manufacturer to impede undesired reactions between calcium and fluoride during storage. However, when the agent is in contact with the saliva the barrier breaks, and the ions are released allowing remineralization [16]. 

Resin infiltration, which is commercially available as Icon(DMG, USA) is a novel, minimally invasive technique, that was initially released to impede the progression of caries in proximal non-cavitated lesions [17]. Its applications were rapidly expanded to include the masking of WSLs of carious and developmental origin [18, 19]. This technique is based on filling the spaces present in hypomineralized enamel with low viscosity resins. Since the resin has a closer refractive index to enamel than water or air, filling the pores with resin leads to masking of the white spot lesion. In addition, the resin will also block diffusion of acids hindering the progression of the lesion [20]. However, the resin infiltrant is mainly composed of TEGDMA, a low molecular weight resin, which may lead to color change. Consumption of food and beverages with different colorants and pigments could lead to discoloration of resin infiltrated enamel over time [21]. This could lead to patient dissatisfaction and frustration. Therefore, color stability is a very important property that must be evaluated for any esthetic treatment.

Clinically, high caries risk patients may require fluoride treatment to help control and impede the progression of WSLs. In addition, resin infiltration is an excellent option to achieve immediate esthetic results to mask these WSLs [22]. Therefore, both treatments may be needed to manage these patients biologically and esthetically. A recent study has studied the effect of combination of both these treatments on physical properties of enamel [23]. Moreover, several studies tested the color restorability and stability of resin infiltration and different remineralizing agents [24–28]. However, up to our knowledge there aren’t any studies that evaluated the color restorability and stability of the combination of both techniques and compared it to each treatment alone. Therefore, in this study we aim to evaluate the effect of resin infiltration, remineralization and their combination on the color restorability of artificially acid demineralized enamel lesions. In addition, we aim to evaluate the color stability of the aforementioned treatments after acid challenge and immersion in common staining beverages. The null hypothesis is that there is no difference when using resin infiltration, remineralization and their combination on the color restorability of artificially acid demineralized enamel lesions. The second null hypothesis is that there is no effect of acidic challenge and beverages on the color stability of artificial acid demineralized enamel lesions subjected to the different treatments used in the study.

## Materials and methods

### Sample size calculation

By adopting an alpha (α) level of (0.05), a beta (β) of (0.2) (i.e. power = 80%), and an effect size (f) of (0.445) calculated based on the results of a previous study [26]; the sample size (n) was found to be a total of (105) samples (i.e. 21 samples per group and 7 samples per subgroup). Sample size calculation was performed using G*Power version 3.1.9.7 2.

### Enamel specimens’ preparation

A total of 53 sound, non-carious and non-restored human posterior teeth were used in this study. Teeth were collected from the department of Oral and Maxillo-Facial Surgery, Faculty of Dentistry, Ain Shams University, under the roles of the Ethical Committee of the Faculty of Dentistry, Ain Shams University (reference number (FDASU-RecED061904) after being extracted for orthodontic purposes. Teeth were cleaned from blood under running water. All soft tissues and hard deposits were removed using a hand scaler (Martin GmbH, Germany). Teeth were then polished with a polishing cup and pumice before preparation procedures. Teeth were examined under stereomicroscope (Nikon, Tokyo, Japan) using RI viewer imaging software (Research instruments, UK), teeth with cracks, stains or lesions were excluded from the study. Teeth were stored in distilled water at 4 °C until use [16, 29]. 

After teeth selection, the roots of the teeth were cut off at the cemento-enamel junction. Each tooth was sectioned mesiodistally under copious amount of coolant using a low speed and a straight hand piece (S 11 LG, W&H, Bȕrmoos, Austria) providing two halves (buccal and lingual) from each tooth to produce a total number of one hundred and five halves (*n* = 105). Complete pulp extirpation with proper irrigation using deionized water was done to ensure complete pulp tissue removal. Each specimen was totally covered with two consecutive coats of clear nail varnish (Amanda, A.R.E) to isolate all specimen surfaces including the sectioned surface, except for a 5 mm^2^ diameter circular window on the middle of the labial or lingual surface of each specimen, that was kept unprotected [30]. Baseline color measurements (L*a*b*) were recorded for all specimens before preparation of white spot lesions.

All specimens were immersed separately in 50 ml demineralizing solution (table 1) for four days except for group 5 (positive control) that was stored in deionized water at room temperature. The solution was not replaced during the entire period of the demineralization of each specimen [31]. After four days, the specimens were removed, rinsed with distilled water for one minute and blot dried with absorbent paper.

**Table 1:** Materials’ composition, manufacturer and lot number

Materials	Composition	Manufacturer and Lot Number
Clinpro™ White Varnish	30–75% pentaerythritol glycerol ester of colophony resin, 10–15% *n*-hexane, 1–15% ethyl alcohol, 1–5% sodium fluoride, 1–5% flavor enhancer, 1–5% thickener, 1–5% food grade flavor, < 5% modified tricalcium phosphate.	3 M ESPE, St. Paul, MN, USA NC59652
ICON^®^	Icon Etch: 15% HCl (hydrochloric acid), pyrogenic silicic acid, surface-active substances. Icon Dry:99% Ethanol. Icon Infiltrant: Triethylene glycol dimethacrylate (TEGDMA) based resin, initiators, additives, stabilizers.	DMG, Hamburg, Germany. 244,373
Demineralizing solution	2.2 mM CaCl2, 2.2 mM KH2PO4, and 50mM Acetic Acid. pH = 4.8	Prepared at Faculty of Dentistry, Ain Shams University
Remineralizing solution	1.5 mM CaCl2, 0.9 mM NaH2PO4, and 0.15 M KCl pH = 7	Prepared at Faculty of Dentistry, Ain Shams University
Artificial Saliva	0.65 g/potassium chloride, 0.058 g/l magnesium chloride, 0.165 g/l calcium chloride, 0.804 g/l dipotassium hydrogen phosphate, 0.365 g/l potassium dihydrogen phosphate, 2 g/l sodium carboxymethyl cellulose and distilled water pH = 7.4–7.8	Prepared at Faculty of Dentistry, Ain Shams University
Coffee	1.8 g of coffee powder into 200 mL of hot water pH = 5.0	Nescafe ^®^ Classic, Nestle, Switzerland
Tea	1 teabag of tea to 200 ml of hot water pH = 4.66–5.04	Lipton^®^, UK

### Treatment protocol

**Fig. 1:**
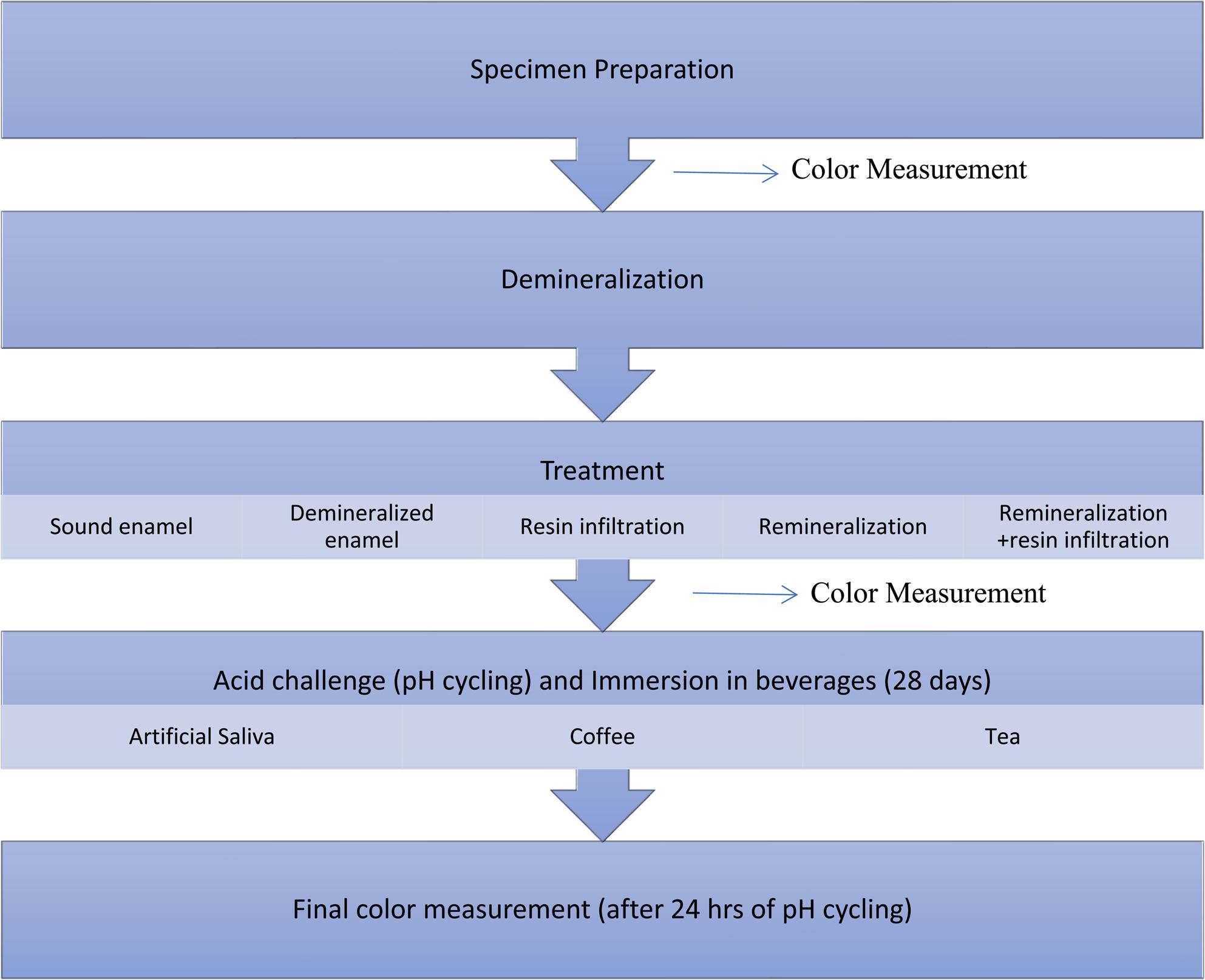
Flow chart showing steps of specimen grouping and measuring intervals

A total of 105 specimens were divided into five groups(*n* = 21) according to the treatment applied to artificially demineralized enamel lesion (figure 1):

#### Group 1 (positive control)

Each specimen of sound enamel was immersed separately in 20 ml of deionized water at room temperature until color measurement. No treatment procedure was performed for the specimens.

#### Group 2 (negative control)

Each specimen of demineralized enamel lesions was immersed separately in 20 ml of deionized water at room temperature until color measurement. No treatment procedure was performed for the specimens.

#### Group 3

Each specimen was removed from deionized water and properly dried with air before the application of resin infiltration to the demineralized enamel lesions. All steps were applied according to the manufacturer’s instructions. The lesions were first etched using 15% HCl (Icon-Etch) for two minutes and were evacuated with a high-volume suction, properly rinsed with oil free water spray for 30 s and air dried after for 30 s. 99% ethanol (Icon-Dry) was applied for one minute and then air dried for 30 s. Finally, the resin infiltrant (Icon-Infiltrant) was applied passively and left to set for three minutes. Any excess beyond the lesion peripheries were removed first with a cotton roll before light curing. The surface was light cured with a LED light curing unit (radii plus, SDI Ltd, Victoria, Australia) with an output of 1500 mW/cm2 for 40 s. A second coat of icon-infiltrant was applied to the lesion, left to set for one minute and light cured for 40 s. The resin infiltrated surface was polished according to manufacturers’ instructions using polishing tips (Shofu, Kyoto, Japan).

#### Group (4)

Fluoride varnish (Clinpro™ White Varnish) was mixed and then applied passively according to the manufacturer’s instructions. Thorough mixing of the content of the 0.5 ml package using micro-brush supplied in the kit was done. A thin layer of varnish was applied passively to the created lesions.

The varnish was left undisturbed for 20 s. Each specimen was immersed separately in artificial saliva for 24 h. After 24 h, the varnish was removed by cotton swab soaked in acetone to ensure removal of all the varnish then washed with distilled water for one minute.

#### Group (5)

Fluoride varnish (Clinpro™ White Varnish) was applied to the specimens as mentioned previously. The varnish was left undisturbed for 20 s and left in artificial saliva for 24 h. After 24 h, the varnish was removed by cotton swab soaked in acetone to ensure removal of all varnish then washed with distilled water for one minute. After removal of the varnish, resin infiltrant (ICON®) was applied according to manufacturer’s instructions as mentioned previously in group 1. 

### pH cycling and immersion in beverages

All specimens of all groups were subjected to pH cycling for 28 days. pH cycling was done by daily immersion of specimens in 20 ml demineralizing solution for 7.5 h followed by immersion in 20 ml of remineralizing solution for 16 h at room temperature. The demineralizing solution was prepared using 2.2 mM CaCl2, 2.2 mM NaH2PO4, and 50 mM acetic acid with pH adjusted to 4.8 (table 1). The remineralizing solution was prepared using 1.5 mM CaCl2, 0.9 mM NaH2PO4, and 0.15 M KCl adjusted to pH 7 (table 1) [32]. Following immersion in pH cycling solutions, specimens were rinsed with distilled water and then were immersed separately in beverages (coffee, tea and artificial saliva as control solution) for 30 min (table 1). The solutions were left to cool to room temperature and were verified by a thermometer before usage [33]. New solutions were prepared daily.

Finally, all specimens were rinsed and stored in deionized water at room temperature before recording final color measurements. (Fig 1).

### Color assessment

The color difference (∆E) was measured according to the Commission International de l’Eclariage (CIE) L* a* b* values, using Cary 5000 Spectrophotometer (Agilent Technologies, NY, USA). The standard illuminant D65 was set, with the reflectance mode and the ultraviolet light included. CIE-Lab color values for each specimen were then calculated by using the color software application which is available through Cary WinUv instrument and supports extensive color calculations and standards. A reference material was used to establish a baseline, and when the standard reference material was being used, the absolute reflectivity of a specimen may be calculated from that of the reference. The specimens were carefully dried with an absorbent paper, not desiccated, and immediately placed over the reflectance port. Because the specimen is smaller in size than what the instrument is designed to measure, a black disk with a small aperture was attached to the front port of the sphere. This allows for exact and repeatable positioning of each specimen and for color measurement under standardized conditions.

All specimens were subjected to color measurement at baseline, after different treatment protocols and after pH cycling and immersion in beverages. The color difference values were measured between the post-treatment and baseline values to assess color restorability, and between the post–pH cycling/staining and post-treatment values to evaluate color stability. A ∆E difference of 0.8 units was considered as a clinical indicator for color change since the 50:50% perceptibility threshold (PT) and acceptability threshold (AT) were reported to be 0.8 and 1.8 [34]. 

Three images were captured for each specimen and the average values for L* (lightness, achromatic color coordinate), a* (green/red coordinate), b* (blue/yellow coordinate) were detected. An online open-source color difference calculator **(**http://colormine.org/**)** was used to calculate the color difference CIEDE2000 (∆E_00_) according to the following equation:

$$\varDelta{E}_{00}=\sqrt{{\left(\frac{\varDelta{L}^{{\prime}}}{{K}_{L}{S}_{L}}\right)}^{2}+{\left(\frac{\varDelta{C}^{{\prime}}}{{K}_{C}{S}_{C}}\right)}^{2}+{\left(\frac{\varDelta{H}^{{\prime}}}{{K}_{H}{S}_{H}}\right)}^{2}+{R}_{T}{\left(\frac{\varDelta{C}^{{\prime}}}{{K}_{C}{S}_{C}}\right)}^{2}{\left(\frac{\varDelta{H}^{{\prime}}}{{K}_{H}{S}_{H}}\right)}^{2}}$$

### Statistical analysis

Numerical data were presented as mean and standard deviation (SD) values. Normality and variance homogeneity assumptions were verified using Shapiro-Wilk’s and Levene’s test respectively. Color restorability data were normally distributed, but the homogeneity assumption was violated so they were analyzed using Welch-one way ANOVA followed by Games-Howell post hoc test. The significance level was set at *p* < 0.05. Color stability data were normally distributed with homogenous variances and were analyzed using one-way ANOVA followed by comparison of simple effects utilizing the error term from the two-way model. P-values were adjusted for multiple comparisons utilizing Bonferroni correction. The significance level was set at *p* < 0.05. Statistical analysis was performed with R statistical analysis treatment version 4.3.1 for Windows1 [35]. 

## Results

### Effect of different treatments on color restorability

There was a significant difference between different treatments (*p* < 0.001). The highest value was found in demineralized enamel (4.41 ± 0.46), followed by remineralization (1.99 ± 0.13), then resin infiltration (1.80 ± 0.21), and remineralization& resin infiltration (0.86 ± 0.12), while the lowest value was found in sound enamel (0.57 ± 0.13) as seen in Table (2). All post hoc pairwise comparisons were statistically significant (*p* < 0.001).

**Table 2:** Intergroup comparisons, mean and standard deviation (SD) values of color restorability (ΔE1) for different treatments

Color restorability (ΔE) (mean ± SD)					
**Resin infiltration**	**Remineralization**	**Remineralization& resin infiltration**	**Demineralized enamel**	**Sound enamel**	*P* value
1.80 ± 0.21^C^	1.99 ± 0.13^B^	0.86 ± 0.12^D^	4.41 ± 0.46^A^	0.57 ± 0.13^E^	<0.001*

Different superscript letters indicate a statistically significant difference within the same horizontal row

*; significant (*p* < 0.05) *ns* non-significant (*p* > 0.05)

### Effect of beverages and different treatments on color stability

Intergroup comparisons, mean and standard deviation (SD) values of color stability (ΔE). for different treatments and beverages are presented in Table (3).

**Table 3:** Intergroup comparisons, mean and standard deviation (SD) values of color stability (ΔE) for different treatments and beverages

Beverage	Color stability (ΔE) (mean ± SD)					*p*-value
	Resin infiltration	Remineralization	Remineralization& resin infiltration	Demineralized enamel	Sound enamel	
Coffee	5.89 ± 0.34^Ca^	8.06 ± 0.38^Ba^	9.05 ± 0.67^Aa^	7.72 ± 0.52^Ba^	4.29 ± 0.21^Db^	< 0.001*
Tea	5.27 ± 0.25^Db^	7.77 ± 0.40^Ba^	8.68 ± 0.39^Aa^	6.94 ± 0.38^Cb^	4.94 ± 0.19^Da^	< 0.001*
Artificial saliva	1.18 ± 0.21^Ac^	0.74 ± 0.08^Bb^	1.18 ± 0.06^Ab^	0.73 ± 0.12^Bc^	0.72 ± 0.08^Bc^	< 0.001*

Uppercase superscript letters indicate a statistically significant difference within the same horizontal row

Lowercase superscript letters indicate a statistically significant difference within the same vertical column

*; significant (*p* < 0.05) *ns* non-significant (*p* > 0.05)

#### Resin infiltration

There was a significant difference between different beverages (*p* < 0.001). The highest value was found in coffee-stained samples (5.89 ± 0.34), followed by tea-stained samples (5.27 ± 0.25), while the lowest value was found at saliva samples (1.18 ± 0.21). All post hoc pairwise comparisons were statistically significant (*p* < 0.001).

#### Remineralization

There was also significant difference between different beverages (*p* < 0.001). The highest value was found in coffee-stained samples (8.06 ± 0.38), followed by tea-stained samples (7.77 ± 0.40), while the lowest value was found at saliva samples (0.74 ± 0.08). Post hoc pairwise comparisons showed saliva samples to have significantly lower value than other beverages (*p* < 0.001).

#### Remineralization& resin infiltration

There was significant difference between different beverages (*p* < 0.001). The highest value was found in coffee-stained samples (9.05 ± 0.67), followed by tea-stained samples (8.68 ± 0.39), while the lowest value was found at saliva samples (1.18 ± 0.06). Post hoc pairwise comparisons showed saliva samples to have significantly lower value than other beverages (*p* < 0.001).

#### Demineralized enamel

There was a significant difference between different beverages (*p* < 0.001). The highest value was found in coffee-stained samples (7.72 ± 0.52), followed by tea-stained samples (6.94 ± 0.38), while the lowest value was found at saliva samples (0.73 ± 0.12). All post hoc pairwise comparisons were statistically significant (*p* < 0.001).

#### Sound enamel

There was a significant difference between different beverages (*p* < 0.001). The highest value was found in tea-stained samples (4.94 ± 0.19), followed by coffee-stained samples (4.29 ± 0.21), while the lowest value was found at saliva samples (0.72 ± 0.08). All post hoc pairwise comparisons were statistically significant (*p* < 0.001).

## Discussion

White spot lesions (WSLs) are considered an esthetic predicament for many patients [7]. Resin infiltration has shown remarkable success in the immediate masking of WSLs [18, 21, 22, 36]. Since fluoride is considered the cornerstone of remineralization therapy, a fluoride-based varnish- Clinpro™ White Varnish – was also used in this study [10]. Nevertheless, clinical situations such as in the case of high caries risk patients may require the combination of both remineralization and resin infiltration techniques. Remineralization with Clinpro™ White Varnish was performed prior to resin infiltration to reduce voids and gaps within the lesion, thereby decreasing the number of pores requiring infiltration and resulting in improved outcomes [23]. To our knowledge, no previous study compared between the color restorability and color stability of Clinpro™ White Varnish and resin infiltration and/ or their combination. Therefore, it was chosen to be investigated in our study.

To imitate the oral environment, a pH cycling model is employed in our study. However, they are incapable of completely replicating the complicated intraoral conditions such as bacterial biofilm, volume of saliva, temperature, and morphology of tooth surface [16, 37]. Therefore, to better reflect clinical situations, daily immersion for 30 min in either coffee, tea or artificial saliva was performed after samples were immersed in pH cycling solutions [32, 38]. Since coffee and tea are both frequently consumed beverages and have a high potential for staining, they were selected in our study [26, 28, 39]. 

Our results revealed that the treatment applied to demineralized enamel lesion had a significant effect on color restorability. In addition, the group combining remineralization and resin infiltration showed the lowest color difference values(ΔE1) between baseline and after treatment and the results were statistically significant from other treatments thus indicating the highest color restorability. When comparing each treatment option alone, resin infiltration group showed significantly lower (ΔE1) compared to remineralization group indicating better color restorability. Therefore, our first null hypothesis was rejected. Apparently, the combination of fluoride remineralization and resin infiltration resulted in a synergistic effect leading to a superior color restorability than each of them alone. The demineralized enamel lesions may have been initially remineralized by the fluoride varnish decreasing the gaps in the demineralized lesion [23]. In addition, fluoride varnish has been shown to improve the smoothness of demineralized lesions by restoring both the prism and interprismatic structures and forming a homogeneous surface, which may, in turn, have positively influenced the optical properties [40]. The resin infiltrant containing TEGDMA which is its main constituent was able to penetrate the remaining gaps present in the lesion, hence masking the lesion [41]. Our results are in agreement with Turska-Szybka et al. [42] In the mentioned randomized clinical study, the effect of resin infiltration in combination with fluoride varnish versus fluoride varnish only was assessed in WSLs on the facial surface of deciduous teeth. The study revealed better results for the resin infiltration and fluoride varnish group in terms of progression of the lesion. However, in the previous study resin infiltration was applied before fluoride application.

The superiority of resin infiltration over fluoride remineralization in color restorability has been reported by many in vitro studies [43–45]. Before applying the resin infiltrant, the hypermineralized surface layer is etched with 15% hydrochloric acid, which has been reported to partially remove approximately 36.70 ± 7.62 μm of the surface [46]. This allows better and deeper penetration of the low viscosity infiltrant into the pores of the subsurface lesions occluding them [41]. On the other hand, remineralization by fluoride varnishes, has been reported to be limited only to the outer 30 μm of the lesion as opposed to the resin infiltrant which was reported to penetrate up to 400 μm of the lesion [47]. Therefore, this surface limited remineralization may not lead to esthetic improvement and masking of the WSL.

Regarding color stability, our results revealed that both the treatment and the beverages have a significant effect. Therefore, our second null hypothesis was rejected. Comparing between treatments, the group combining remineralization and resin infiltration resulted in the highest color differences (ΔE2) indicating the lowest color stability while resin infiltration resulted in the lowest values indicating highest color stability and the results were statistically significant among all beverages. Moreover, all values exceeded the CIEDE2000 (ΔE_00_) 50:50% perceptibility threshold (PT) and acceptability threshold (AT) which were reported to be 0.8 and 1.8 respectively [34]. Regarding the remineralization combined with resin infiltration group, the acid challenge in addition to the beverages has seemingly affected the enamel structure of the remineralized and resin-infiltrated enamel. According to a recent study, when the resin penetrates the WSL, the enamel crystallites become enveloped with resin forming a hybrid layer. This resin embedded enamel was found to be more acid resistant [41]. However, in the remineralization combined with resin infiltration group, the enamel was initially remineralized. Therefore, this hybrid layer may have failed to form with the remineralized enamel leading to less acid resistance. Furthermore, the acid challenge may have led to surface mineral loss in addition to roughness which probably led to the adherence of stains and discoloration [48]. 

The lower color stability of remineralization compared to other treatments may be attributed to incomplete remineralization of the lesion and presence of porous enamel [49]. The acid challenge combined with the beverages may have also led to further mineral loss which may have enhanced the staining. Another factor which may have contributed to the findings is the short period between application of fluoride varnish and acid challenge and immersion in beverages. This short gap was probably not enough for sufficient remineralization to have occurred, which reflected on our results regarding both color restorability and stability.

In line with our findings, AH Ayad et al. [50], concluded that resin infiltration was more color stable than remineralization using CPP-ACP. The low viscosity resin can deeply infiltrate into the demineralized lesion by capillary action and therefore occluding the pores and blocking pathways for chromophores. On the contrary, Cohen-Carneiro et al. [26], concluded that remineralization with fluoride was more color stable than resin infiltration after using coffee and wine as staining solutions. The reasons for lower color stability of resin infiltration were attributed to the TEGDMA which is the main constituent of the resin infiltrant. This monomer is known for its hydrophilicity and high water sorption which naturally may lead to increased tendency for staining. Nevertheless, different fluoride remineralization agents, protocols for staining and timings of color measurements were used in these studies compared to our study.

Regarding beverages, both coffee and tea caused significant color changes among all groups. Coffee and tea are both heavily consumed beverages with a strong discoloration tendency. However, our results show stronger discoloration for coffee than tea in all groups except for positive control group where the tea showed greater discoloration than coffee. Although coffee exhibited higher values, the difference between coffee and tea was not statistically significant in either the remineralization group or the remineralization and resin infiltration group. Similarly, Rey at al. [39], also reported that both tea and coffee caused significant staining to resin infiltration samples and that coffee showed a higher staining effect than tea. On the contrary, Alqahtani al. [51], reported that tea had a stronger staining effect than coffee. Generally, both tea and coffee contain yellow colorants. However, tea was reported to cause discoloration through surface adsorption of colorants [39, 51]. Coffee, on the other hand, causes discoloration thorough surface adsorption and absorption of colorants. Coffee pigments were also reported to have high affinity to the resin polymer network facilitating penetration of the pigments [30].

There are several limitations present in our study. Initially, it is an in vitro study. Therefore, results must be interpreted with caution and cannot be extrapolated to the much more complex clinical conditions. In addition, the demineralized enamel lesions were artificially created which may not replicate the naturally occurring WSLs. Furthermore, the depth of the demineralized lesions was not assessed to confirm whether subsurface lesions had developed.

## Conclusions

Within the limitations of the study, our results revealed that remineralization preceding resin infiltration had a positive impact on color restorability whilst they showed the least color stability. Resin infiltration alone was the most color stable among the treatments. Both coffee and tea have a strong staining potential and negatively affect color stability of remineralization and resin infiltration.

## Data Availability

The datasets generated and/or analyzed during the current study are available from the first author upon request.
